# Challenges Associated With Rural‐Urban Stratification for Generalizing Birth Outcomes: Insights From the ECHO Cohort

**DOI:** 10.1111/jrh.70163

**Published:** 2026-05-12

**Authors:** Daniel Beene, Debra A. MacKenzie, Carlos A. Camargo, Rana F. Chehab, Amy J. Elliott, Griffith Gao, Kelly A. Hirko, Margaret R. Karagas, Sarah Keim, Lacey A. McCormack, Alicia K. Peterson, Hananeh Sadeghi, Nissa Towe‐Goodman, Amii M. Kress, for the ECHO Cohort Consortium

**Affiliations:** ^1^ Department of Epidemiology Johns Hopkins Bloomberg School of Public Health Baltimore Maryland USA; ^2^ College of Pharmacy University of New Mexico Health Sciences Center Albuquerque New Mexico USA; ^3^ Department of Emergency Medicine Massachusetts General Hospital Harvard Medical School Boston Massachusetts USA; ^4^ Division of Research Kaiser Permanente Northern California Pleasanton California USA; ^5^ Avera Research Institute Avera McKennan Hospital & University Health System Sioux Falls South Dakota USA; ^6^ College of Engineering Northeastern University Boston Massachusetts USA; ^7^ Department of Epidemiology and Biostatistics College of Human Medicine Michigan State University East Lansing Michigan USA; ^8^ Department of Epidemiology Geisel School of Medicine at Dartmouth Hanover New Hampshire USA; ^9^ Center for Biobehavioral Health Research Institute at Nationwide Children's Hospital and The Ohio State University Columbus Ohio USA; ^10^ Frank Porter Graham Child Development Institute The University of North Carolina at Chapel Hill Chapel Hill North Carolina USA

**Keywords:** cohort studies, ECHO Cohort, generalizability, preterm birth, rural–urban classification

## Abstract

**Purpose:**

Efforts to generalize findings from the Environmental influences on Child Health Outcomes (ECHO) Cohort across rural and urban areas are challenged by limitations in both sample composition and the classification schemes used to define place. We evaluated how rural‐urban stratification affects the interpretation and generalizability of preterm birth (PTB) prevalence proportions in the ECHO Cohort compared to national benchmarks.

**Methods:**

We used a population data science approach to compare bootstrap estimates of PTB prevalence in ECHO (2017–2019, 2020–2022) to county‐level prevalence from the National Center for Health Statistics, stratified by rural–urban classification (RUCC, UIC, NCHS), race/ethnicity, education, and income. We applied post‐stratification weights and conducted sensitivity analyses.

**Findings:**

Overall PTB prevalence in ECHO was statistically similar to that in US live births. Estimates varied by rural–urban classification scheme but showed no consistent directional difference. Stratifying by race and education revealed variability in PTB differences and gaps in subgroup representation within the analytic sample. Post‐stratification increased PTB estimates slightly and stabilized rural estimates. Two predominantly rural cohort sites strongly influenced rural means; excluding one reversed the direction of rural–urban difference while excluding the other increased it. Supplemental analyses showed regional variability in PTB prevalence and suggested that living above 130% of the federal poverty level may be protective.

**Conclusions:**

Rural–urban stratification alone, without accounting for the context of rural places, limits generalizability and may obscure differences between samples drawn from large cohort studies and the broader population. Context‐aware stratification may improve validity and equity in population health research.

## 1 Introduction

Rurality is a commonly used but inconsistently defined construct in population health research, often operationalized through geographic codes that serve as proxies for the social and structural context and composition of rural places [1, 2]. Despite rurality being defined and categorized in many different ways, a vast body of research suggests that rural populations in the United States experience disparities in health outcomes and the provision of health services [3, 4, 5], potentially stemming from diminished access to services, higher poverty rates, aging populations, and unique environmental exposures [6, 7, 8, 9, 10, 11, 12]. Furthermore, racial and ethnic minorities living in rural areas may experience disproportionate adverse effects of weathering on health outcomes [13, 14].

Many measures, including certain sociodemographic indicators we explore here, may fall along rural–urban lines, but the degree to which they are causally relevant is inconsistent [4]. Building on prior work examining rural–urban classification in the Environmental influences on Child health Outcomes (ECHO) Cohort [4], the present study focuses on the effect of rural–urban stratification in inferential comparisons. McCormack et al. [4] highlighted how collapsing rural–urban codes into binary categories can obscure variation in prevalence estimates of childhood obesity. We extend this line of inquiry by evaluating how rural–urban stratification affects the generalizability of estimated differences in preterm birth (PTB) prevalence between ECHO and national benchmarks.

Understanding differences in PTB prevalence across the rural–urban divide may provide useful insight into how the within‐group heterogeneity of both rural and urban places affects measurement of health outcomes. Statistical challenges in measuring PTB rates in rural counties stemming from numerical imbalance, urban sampling biases, and smoothing of estimates notwithstanding [15], research suggests that PTB rates in rural counties are influenced by medical vulnerability and limited perinatal care [16]. Research also shows that while PTB rates were historically similar across the rural–urban gradient [17] despite limited access to physician intervention [18], more recent losses to obstetric services in rural areas are significantly associated with increased rural PTB rates [19]. Meanwhile, socioeconomic factors irrespective of geography are frequently implicated in PTB rates [20].

The aim of the present study is to evaluate whether rural–urban differences in PTB prevalence observed in the ECHO Cohort reflect true geographic variation or are driven by cohort site composition in a study not designed to estimate rural–urban contrasts. Most rural ECHO participants have been recruited from a small number of cohort sites with different population compositions and rural contexts, complicating interpretation of rural–urban differences. By comparing ECHO PTB percentages to national county‐level estimates and applying sensitivity analyses, we assess the extent to which findings from ECHO can be generalized across place‐based contexts.

## 2 Methods

The ECHO Cohort is a prospective prenatal, birth, and pediatric cohort comprising 69 cohort sites as of October 2023 [21]. For the participants included in the present analysis, recruitment into the ECHO‐Wide Cohort Data Collection Protocol predominantly occurred during pregnancy, though some children were enrolled after birth [21]. Participants were drawn from all 50 states, Washington DC, and Puerto Rico; however, consistent with the vast majority of research to date, rural participants were not recruited according to their proportion of the population across regions (nor was ECHO designed to be a nationally representative sample). Due to the challenges inherent in research within rural populations, few cohort sites enrolled rural participants; these sites themselves had unique compositions and rural contexts. Generalizing findings to the broader population thus necessitates careful consideration of underlying socioeconomic, demographic, environmental, and other structural characteristics of the analytic population.

The analytic sample is composed of 13,330 births from 13,150 pregnancies from the ECHO Cohort in which the child was born between 2017 and 2022, for whom high‐quality geocodes of residential address at the time of delivery were available, and who were not enrolled through a PTB‐enriched cohort site. Multiple gestation pregnancies were included in the analytical sample to reflect the National Center for Health Statistics (NCHS) Birth Files methodology for estimating PTB prevalence percentages [22]. The present study was approved by the Institutional Review Board and Tribal Human Research Review Board (where applicable). Participants provided informed consent. Figure S1 shows a flowchart of the study participants according to the inclusion/exclusion criteria and Table S1 lists the proportion of eligible ECHO participants with high‐quality geocodes stratified by rural and urban.

The outcome of interest was binary PTB harmonized across cohort sites as a gestational age of less than 37 completed weeks at delivery. Covariates included county‐level rural–urban classifications measured using the USDA Rural–Urban Continuum Codes (RUCC) for both 2013 and 2023, Urban Influence Codes (UIC) for 2013, and the National Center for Health Statistics (NCHS) Urban–Rural Codes (URC) for both 2013 and 2023. Additionally, maternal highest educational attainment, race and ethnic identity, and household income were considered. Missing observations in education and race/ethnicity (21.5% and 3.5%, respectively) were imputed using the MICE package in R (Version 4.4.3). Because of a high degree of missingness of household income (64.7%) and because of broad harmonized categories, a binary flag for households above or below 130% the federal poverty level (FPL) was explored as a secondary analysis on a subset of the analytic sample (*N* = 4239).

County‐level PTB prevalence proportions in the analytic sample were compared with county‐level national PTB percentages derived from the NCHS Birth Files compiled through the Vital Statistics Cooperative Program [23] and linked to counties via the Health Resources and Services Administration (HRSA) Maternal and Infant Mapping Tool, which provides estimates of births in counties with a small number of births or where data are otherwise suppressed [24, 25]. PTB percentages are available as cumulative 3‐year estimates of PTB risk aggregated from 2017 through 2019 and 2020 through 2022. We refer to the PTB prevalence throughout as the county‐level proportion of live births occurring before 37 completed weeks’ gestation.

### 2.1 Statistical Analysis

We used a population data science approach [26, 27, 28] to evaluate mean differences in county‐level PTB prevalence between the ECHO Cohort and national benchmarks. Rather than relying on parametric assumptions, we applied nonparametric bootstrap resampling across 10,000 iterations to empirically estimate sampling distributions of PTB prevalence within strata of interest. National estimates were compared to bootstrap distributions, and a difference was considered meaningful if the national benchmark lay outside the 95% bootstrap percentile interval. This approach allowed us to identify instability driven by small subgroups or influential cohort sites.

We compared differences in the sociodemographic composition of the analytical sample to 5‐year estimates of the US population at childbearing age from the 2022 American Community Survey (ACS) by comparing proportions of racial/ethnic and educational attainment categories by rural and urban classifications [29, 30]. Race/ethnicity and education were used as sociodemographic indicators of structural context with strong coverage in both ECHO and national data. Next, counts of children in the sample in each rural–urban classification level were compared to the US population aged 5 and younger [31] using chi‐squared tests.

Because participants in the analytic sample resided in 460 out of 3244 US counties and county equivalents, we evaluated differences in PTB prevalence using a bootstrap approach to directly match counties between populations across 10,000 replicates. For each bootstrap replicate, we randomly selected one completed dataset from the multiple imputation set and drew approximately 67% of the ECHO sample with replacement. Each sampled ECHO participant was then matched to county‐level PTB rates by county and birth‐year group (2017–2019 or 2020–2022) to account for temporal variation in population risk. We then dichotomized samples into rural or urban classifications and compared group means using *t*‐tests. Additionally, we plotted the differences between mean county‐level PTB prevalence and mean PTB prevalence from the analytic sample as the US county‐level mean minus the ECHO county‐level sample mean and computed 95% confidence intervals for each distribution. We recoded rural–urban dichotomies for each classification scheme as follows:

- RUCC (2013 and 2023): Rural/non‐metro RUCC codes 4–9; Urban/metro RUCC codes 1–3
- UIC: Rural/non‐metro UIC codes 3–12; Urban/metro UIC codes 1–2
- NCHS‐URC (2013 and 2023): Rural/micropolitan NCHS codes 5–6; Urban/metro NCHS codes 1–4.

We ran bootstrap analyses in five main and two supplemental models. First, we performed a full unadjusted model to establish a baseline overall difference between the ECHO Cohort sample and the United States. Second, we stratified the unadjusted model by rural and urban classifications and plotted the distribution of differences in group means. Third, we conducted a fully stratified model comparing group means by six race/ethnicity strata (Hispanic, American Indian/Alaska Native (AIAN), Black, Asian, White, and other/two or more races (non‐Hispanic)) and five educational attainment strata (less than a high school diploma, high school diploma or equivalent, some college, bachelor's degree, graduate degree) by rural or urban classification, for a total of 60 paired strata.

Next, we calculated post‐stratification weights based on the joint distribution of race/ethnicity and educational attainment using 2022 ACS 5‐year estimates. We derived weights by comparing the proportion of each race‐education stratum in the national population to its corresponding proportion in the ECHO analytic sample. We then applied the weights during bootstrap resampling, stratified by rural and urban residence, to partially account for observed sociodemographic differences across groups. We additionally conducted two sensitivity analyses. First, we re‐estimated the primary post‐stratified bootstrap model to only include one observation per pregnancy to address potential nonindependence among multiple gestation pregnancies. Second, we conducted a leave‐one‐out analysis using the post‐stratified bootstrap model, excluding one cohort site per iteration to assess the influence of individual cohorts on estimated differences. In each iteration, we evaluated the influence of the excluded cohort site by comparing the weighted difference in group means between ECHO and US PTB prevalence proportions.

Additionally, in one supplemental analysis we stratified the bootstrap sample by rural or urban counties within census regions (West, South, Midwest, and Northeast, excluding Puerto Rico due to sample size). In the second supplemental analysis, we stratified the analytical sample subset with complete household income data by households living either below or greater than or equal to 130% of the FPL in rural or urban counties. Both supplemental analyses used post‐stratification sampling weights. All analyses were conducted in R (Version 4.4.0).

## 3 Results

**FIGURE 1:**
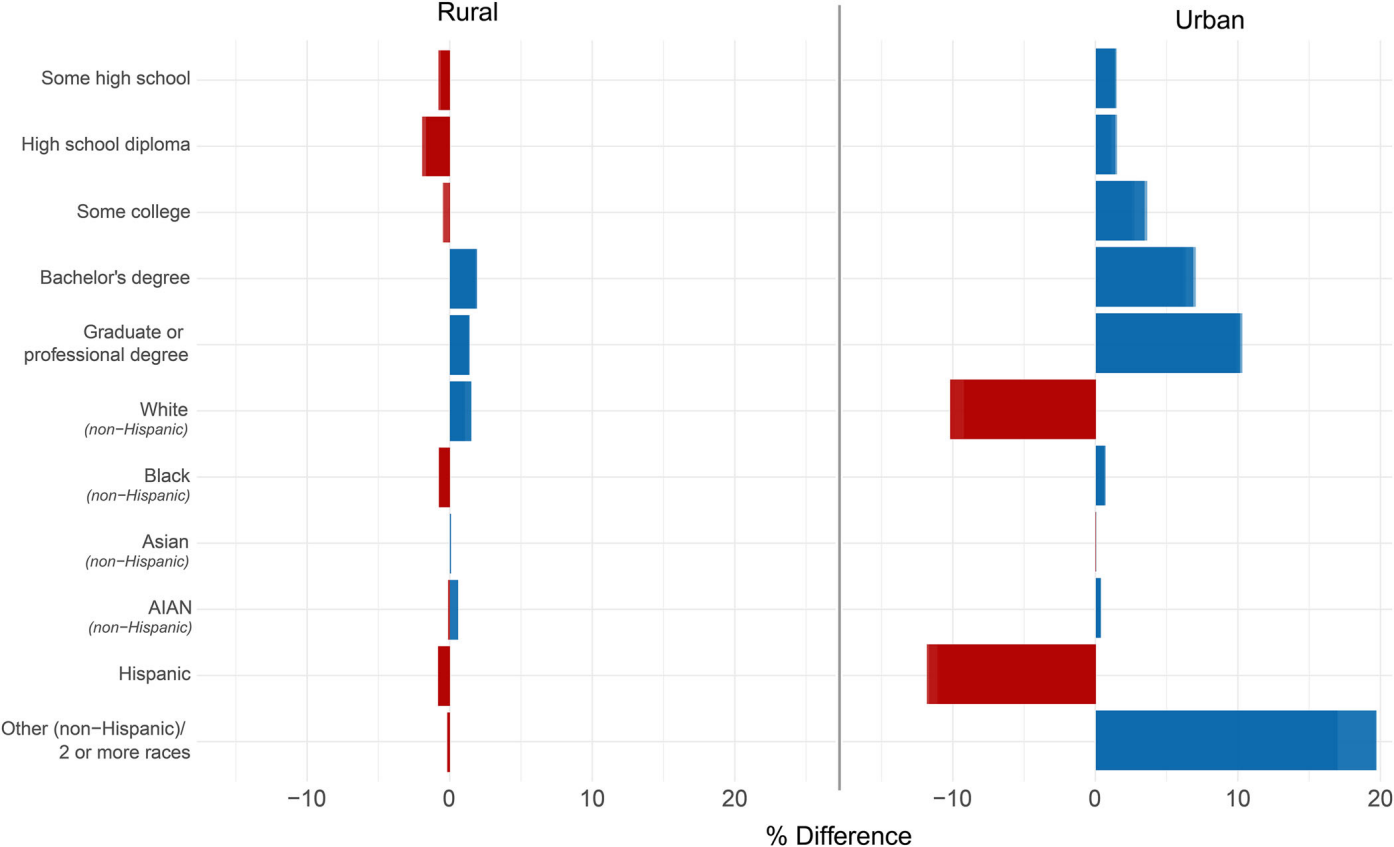
Differences between the analytic sample and US estimates in educational attainment and race/ethnicity categories stratified by rural and urban counties. Positive (blue) indicates the analytic sample is higher and negative (red) indicates the analytic sample is lower than the US population.

Table 1 presents summary statistics of the analytical sample prior to imputation. The comparison between the analytic sample and US population of childbearing age by sociodemographic characteristics showed that, in general, there were fewer Hispanic and non‐Hispanic White participants and more mixed‐race (non‐Hispanic) participants in urban counties in the analytic sample than national averages, as well higher overall rates of college degrees in the analytic sample in both urban and rural counties. All other racial and educational attainment categories demonstrated differences of less than 2.75% in both rural and urban counties (Figure 1).

**TABLE 1:** County‐level birthing parent educational attainment and child race/ethnicity by rural or urban classification in the analytic sample.

	RUCC 2013^a^		RUCC 2023^a^		UIC 2013^b^		NCHS‐URC 2013^c^		NCHS‐URC 2023^c^	
	*Rural*	*Urban*	*Rural*	*Urban*	*Rural*	*Urban*	*Rural*	*Urban*	*Rural*	*Urban*
*n*	1909	11,421	1909	11,421	1909	11,421	1908	10,953	1908	10,953
**Birthing parent highest education**										
Some high school	0.37%	8.21%	0.15%	8.30%	0.37%	8.21%	0.37%	8.14%	0.15%	8.23%
High school diploma	1.21%	16.54%	0.86%	16.58%	1.21%	16.54%	1.21%	15.84%	0.85%	15.88%
Some college	2.94%	21.15%	2.38%	21.30%	2.94%	21.15%	2.93%	19.73%	2.30%	19.95%
Bachelor's degree	3.79%	21.76%	3.61%	21.90%	3.79%	21.76%	3.79%	20.66%	3.58%	20.83%
Graduate or professional degree	2.53%	21.49%	2.41%	21.60%	2.53%	21.49%	2.53%	21.02%	2.35%	21.19%
**Child race/ethnicity**										
White (non‐Hispanic)	12.57%	34.08%	11.89%	34.77%	12.57%	34.08%	12.57%	34.08%	11.89%	34.77%
Black (non‐Hispanic)	0.07%	11.79%	0.06%	11.80%	0.07%	11.79%	0.07%	11.79%	0.06%	11.80%
Asian (non‐Hispanic)	0.22%	5.78%	0.22%	5.78%	0.22%	5.78%	0.22%	5.78%	0.22%	5.78%
AIAN (non‐Hispanic)	1.04%	0.85%	0.12%	0.79%	1.04%	0.85%	1.04%	0.85%	0.12%	0.79%
Hispanic	0.21%	2.92%	0.19%	2.94%	0.21%	2.92%	0.21%	2.92%	0.19%	2.94%
Other (non‐Hispanic)/2 or more races	0.38%	30.09%	0.49%	29.94%	0.38%	30.09%	0.37%	26.47%	0.33%	26.47%

Abbreviations: AIAN, American Indian/Alaska Native; NCHS‐URC, National Center for Health Statistics Urban–Rural Codes; RUCC, Rural–Urban Continuum Codes; UIC, Urban Influence Codes.

*Note*: NCHS codes not available for Puerto Rico: some participants are excluded.

^a^ Rural/non‐metro RUCC codes 4–9; Urban/metro RUCC codes 1–3.

^b^ Rural/non‐metro UIC codes 3–12; Urban/metro UIC codes 1–2.

^c^ Rural/micropolitan NCHS codes 5–6; Urban/metro NCHS codes 1–4.

Chi‐square tests indicated that, for every rural–urban classification scheme, the distribution of children in the study sample differed significantly from the national population distribution across classification levels (Table 2). In all schemes, large positive residuals indicated that the most urban classification (class 1) was overrepresented in the ECHO sample, while small or mid‐sized metro classifications (typically classes 2 or 3) were the most underrepresented. Rural classifications showed smaller and more variable differences, with some levels slightly overrepresented.

**TABLE 2:** Chi‐squared test results between the distribution of ECHO children in the analytic sample born from 2017–2022 and the distribution of the estimated 2022 US population aged <5^a^ by rural–urban classification scheme.

Classification scheme	*X* ^2^	df	*p*‐val	Residuals by class (urban/metro to rural/non‐metro)											
				1	2	3	4	5	6	7	8	9	10	11	12
RUCC (2013)	3829	8	2.2e‐16^**^	19.62	−42.65	12.80	−13.71	14.89	−15.40	23.06	1.16	16.76	—	—	—
RUCC (2023)	2538.9	8	2.2e‐16^**^	18.64	−25.31	−8.29	−14.09	12.35	−15.69	27.99	−6.08	8.02	—	—	—
UIC (2013)	3818.6	11	2.2e‐16^**^	19.62	−28.76	−10.20	−8.27	−14.61	−5.44	−1.79	46.17	−4.85	2.22	−4.35	−3.00
NCHS‐URC (2013)	5375.6	5	2.2e‐16^**^	50.05	−26.44	−42.43	9.40	13.51	−10.01	—	—	—	—	—	—
NCHS‐URC (2023)	3992.5	5	2.2e‐16^**^	48.46	−24.84	−25.37	−11.72	9.58	−12.42	—	—	—	—	—	—

Abbreviations: AIAN, American Indian/Alaska Native; NCHS‐URC, National Center for Health Statistics Urban–Rural Codes; RUCC, Rural–Urban Continuum Codes; UIC, Urban Influence Codes.

** Significant at *p* < 0.001.

^a^ American Community Survey 5‐Year Estimates table B01001—Sex by Age.

**FIGURE 2:**
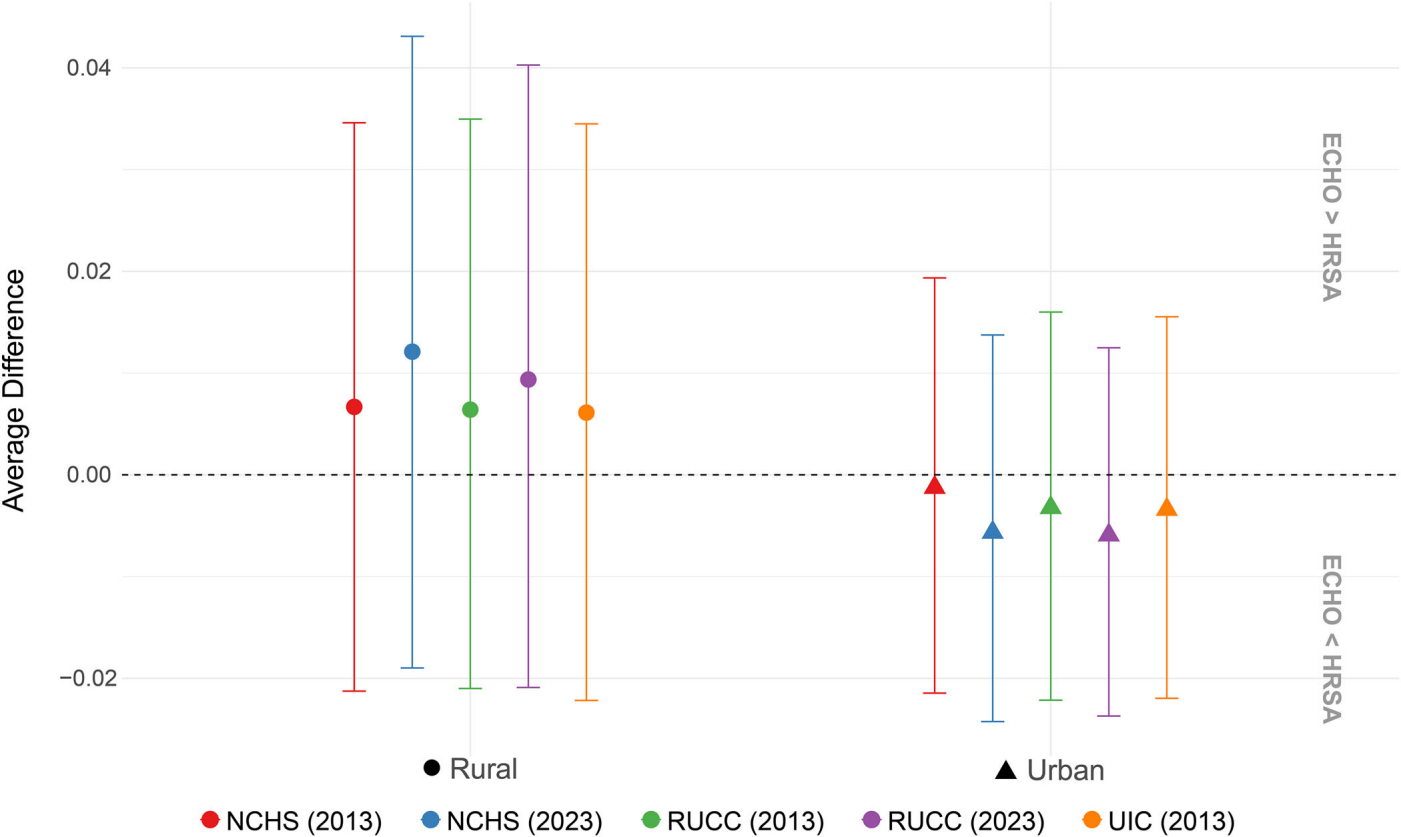
Average difference in 2017–2022 county‐level PTB rates by rural/urban classification, calculated as the analytic sample mean PTB minus US mean PTB.

The full unadjusted bootstrap model indicated no significant differences between ECHO and US PTB prevalence overall, with a moderate difference of only 0.001% ± 0.008% among all sampled counties. The unadjusted model stratified by rural or urban counties for all five classification schemes revealed moderately higher rural PTB prevalence in ECHO and confidence intervals for bootstrap replicates were generally narrower for urban than rural counties (Figure 2). Additionally, fewer than 4% of bootstrapped *t*‐tests were statistically significant (*p* < 0.05), with confidence intervals overlapping zero across all classification schemes.

**FIGURE 3:**
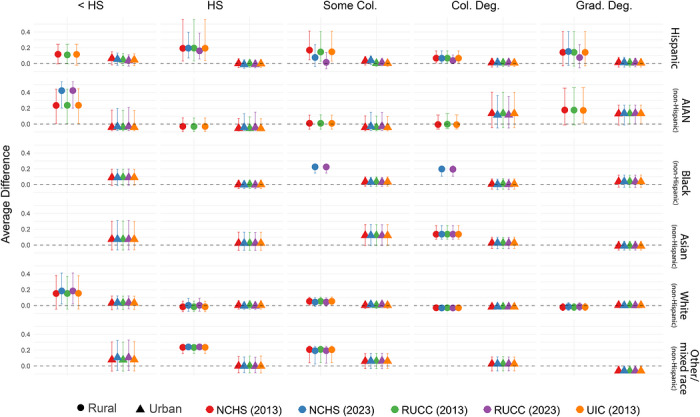
Average difference in 2017–2022 county‐level PTB rates by rural/urban classification, stratified by race/ethnicity and birthing parent educational attainment.

We did not observe any clear trend of over‐ or underestimation of PTB prevalence in the ECHO Cohort compared to the United States in strata paired by race/ethnicity and educational attainment (Figure 3). No strata demonstrated significance in more than 95% of *t*‐tests. A total of 10 racial/educational attainment groups were not observed in the analytical sample, all in rural groups. Specifically, the analytical sample did not include rural participants with less than a high school education who are Black or Asian, or other/mixed race; rural participants with a high school diploma who are Black or Asian; rural participants with some college education who are Asian; rural participants with a college degree who are other/mixed race; or rural participants with a graduate or professional degree who are Black, Asian, or other/mixed race.

**FIGURE 4:**
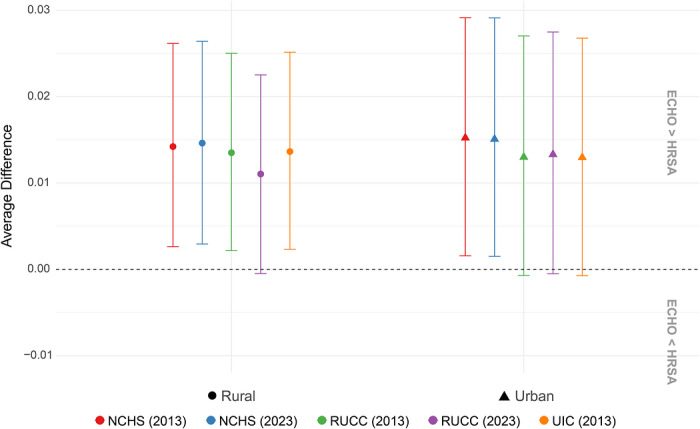
Average difference in 2017–2022 county‐level PTB rates by rural/urban classification, post‐stratified using sample weights for race/ethnicity and education.

After post‐stratification, ECHO PTB prevalence proportions were slightly higher than US estimates, ranging from 0.012% ± 0.007% to 0.015% ± 0.007% in urban counties and from 0.011% ± 0.006% to 0.015% ± 0.006% in rural counties. Confidence intervals for rural counties classified using 2023 RUCC codes slightly overlapped 0, as did confidence intervals for urban counties classified using 2013 and 2023 RUCC codes and 2013 UIC codes (Figure 4). We further examined post‐stratified estimates by region (Figure S2) and found that there were no ECHO participants in the analytic sample in rural counties in the South. Participants living in the rural Midwest had the smallest confidence intervals due to the large number of rural participants there, as well as a mean PTB prevalence consistently higher than that of the United States. Similarly, participants living in urban Northeast, South, and West counties mean PTB prevalences larger than those in the United States, excluding urban Northeastern counties classified using NCHS and 2023 RUCC codes, though confidence intervals overlapped zero for all classification schemes. Conversely, participants living in urban midwestern and rural northeast and west counties all had mean PTB prevalences smaller than those in the United States, though most confidence intervals overlapped zero. There were too few rural observations in Puerto Rico to draw inferences about rural–urban differences in PTB prevalence.

Analysis of the post‐stratified subset stratified by households’ annual income suggests that households below 130% of the FPL were more likely to demonstrate higher PTB prevalence than national norms, regardless of urban or rural county residence (Figure S3). Confidence intervals were centered near 0 and narrower among households above 130% FPL (*n* = 3325) and consistently positive but wider with more variable point estimates among those below 130% FPL (*n* = 914).

The pregnancy‐level sensitivity analysis using post‐stratified sampling weights produced effect estimates in the same direction and of similar magnitude across all rural–urban schemes. Estimates were slightly more negative under pregnancy‐level resampling (absolute shift ≈0.002–0.006), with overlapping confidence intervals and no substantively different conclusions (Table S2).

**FIGURE 5:**
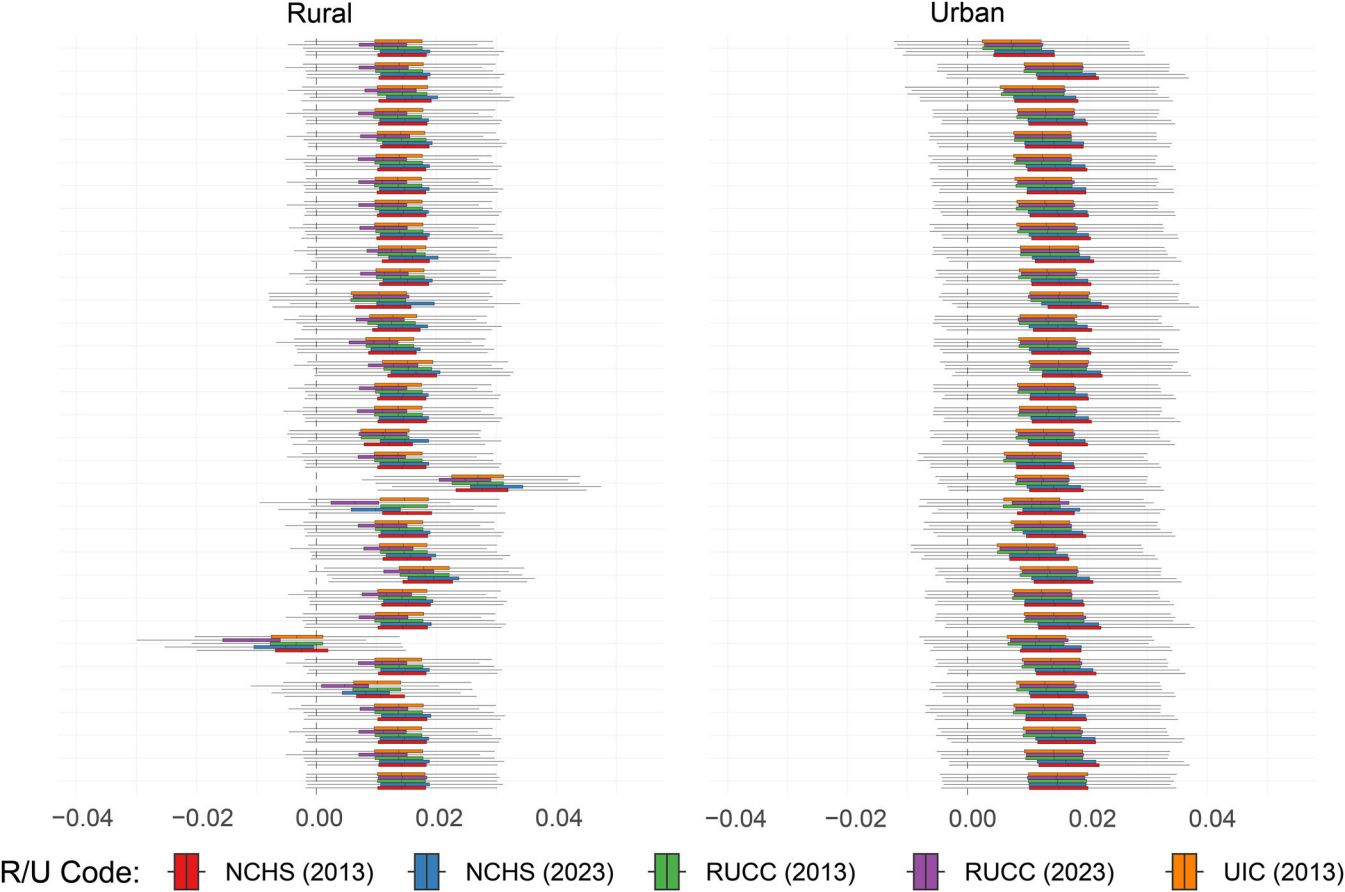
Average differences in mean PTB rates between the analytic sample and the United States using post stratified sample weights, dropping one cohort per bootstrap run.

The sensitivity analysis dropping one cohort site with post‐stratified weights per bootstrap run revealed in general slightly higher mean PTB prevalence percentages in ECHO compared to the United States with most confidence intervals overlapping 0 (Figure 5). Estimates in urban counties were relatively stable across all classification schemes in all sensitivity iterations. Estimates were more variable in rural counties with two notable large differences when predominantly rural cohort sites were excluded. Specifically, when one largely rural low‐risk cohort site was removed, mean PTB prevalence in rural counties increased by 0.025% ± 0.006% to 0.03% ± 0.007% with all confidence intervals greater than 0. When another largely rural cohort site with a high‐risk population was removed from analysis, mean PTB prevalence decreased to less than national means by 0.002% ± 0.006% to 0.011% ± 0.007% in rural counties only, although interquartile ranges or confidence intervals overlapped 0 for all classification schemes.

## 4 Discussion

While cohort composition in large cohort studies may potentially bias exposure and outcome estimates, it does not necessarily affect estimates of exposure‐outcome associations [32]. However, our findings demonstrate that structural differences can meaningfully shape population‐level estimates when stratified by rural–urban classification. This finding underscores the importance of critically evaluating how urban and rural categories are operationalized and interpreted, especially when they are used as proxies for contextual factors relevant to the relationship between place and birth outcomes, such as access to perinatal care. For all rural–urban codes, the study sample is statistically dissimilar in terms of national educational attainment and race/ethnicity among similar age groups. However, rather than focusing on potential sampling bias, these sociodemographic comparisons provide important insight into the structure of the analytic population, telling us which groups are more or less influential in our estimates. Specifically, ECHO participants included in the analytic sample tended to be more likely to have a bachelor's degree or higher than the US population, although this difference is less notable in rural regions. While there were only slightly fewer rural sample participants whose highest level of educational attainment is a high school diploma, this may influence PTB estimates stratified by race and ethnicity, which show large differences in that educational stratum.

Nationally, the study sample demonstrated a statistically similar PTB proportion to the United States, which suggests that taken as a whole, the ECHO Cohort is not necessarily a low‐ or high‐risk sample. Across 10,000 bootstrap replicates of the unadjusted model, we consistently observed similarities in PTB prevalence between ECHO and the United States in both rural and urban strata. However, the direction of the mean PTB point estimates differed across rural–urban classification schemes, with rural counties showing slightly higher PTB prevalence and urban counties suggesting the opposite. Slight variability in point estimates may reflect more than random variation and instead suggest that certain places do not fit cleanly into rural or urban categories that are sensitive to how those categories are defined.

The instability in PTB point estimates across rural–urban classification schemes may further reflect methodological differences and deeper conceptual tension in categorizing places at the rural–urban margin. As Sharma‐Wallace [33] argues, the rural–urban interface is a spatially and socially complex zone that deserves its own environmental justice inquiry. These marginal geographies are often situated at the edges of metropolitan influence or within peripheral zones of state investment where people may bear the compounded effects of limited infrastructure, racialized land use legacies, and environmental burdens [9, 34, 35, 36]. The health risks observed in these areas may emerge through (bio)political and economic processes that shape uneven access to care, exposure to environmental hazards, and community resilience [37, 38]. The observed variation highlights the limitations of relying solely on rural–urban codes and suggests the need for relational, context‐sensitive measures that reflect the lived realities of place [34].

Similarly, we cannot directly extrapolate that similarities between the ECHO Cohort and the United States exist in spite of the different distribution of ECHO participants by rural or urban counties. When we adjusted for sociodemographic differences between the ECHO Cohort and the US population, point estimates of differences in PTB prevalence percentages between the ECHO analytic sample and the United States increased. These observed differences partially reflect the underlying composition of the ECHO Cohort, which includes a larger proportion of urban participants relative to the US population. While not a source of sampling bias, this structure influences the interpretation of population‐level comparisons and highlights the importance of analytic strategies that account for variation in cohort composition. Incidentally, noting the need for more pediatric research among rural populations, the ECHO program has co‐developed the ECHO IDeA States Pediatric Clinical Trials Network (ISPCTN) with the National Institutes of Health (NIH) to increase access among underrepresented rural populations to clinical trials through ECHO [39].

Unobserved strata in the analytic sample (specifically, those defined based on race/ethnicity and educational attainment) do not necessarily reflect missingness in the full ECHO Cohort. This study required high‐quality geocoded residential data for inclusion, and not all ECHO participants met that criterion. As a result, the composition of the analytic sample may differ from the broader cohort. In future studies, this type of geospatial inclusion criterion may compound selection effects when combined with rural–urban stratification.

The observed regional variation in post‐stratified PTB estimates suggests that the ECHO Cohort's generalizability varies across the United States. The absence of rural Southern participants in the sample limits interpretation in a region with known rural health disparities [40]. High PTB prevalence proportions in the rural Midwest, driven by a single high‐risk cohort, demonstrate how individual samples can shape broader patterns. Conversely, lower PTB prevalence but wide confidence intervals in the rural West underscore the need for caution when interpreting estimates from small or less diverse samples. Stratifying by household income revealed that poverty was a more consistent marker of elevated PTB risk than rural–urban classification: households below 130% of the FPL demonstrated higher PTB prevalence relative to national estimates regardless of rural or urban county residence, while estimates among households above 130% FPL were closer to national norms and more precise. These results suggest that income stratification helps illustrate population‐level PTB risk patterns that rural–urban codes alone do not consistently capture.

The leave‐one‐cohort‐out sensitivity analysis indicates that a single cohort site with a relatively large rural sample in the Midwest substantially influenced the observed higher mean PTB prevalence in rural counties. In contrast, removing a predominantly rural cohort site in the Northeast yielded the opposite pattern, suggesting that rural participants across cohort sites may reside in distinct contextual environments that differentially shape birth outcomes. These findings highlight the challenge of generalizing cohort study results using rural–urban classifications when rural subsamples are not only smaller but also structurally heterogeneous, which can amplify the overall influence of individual cohort sites. In this way, rural–urban stratification in unbalanced samples from cohort studies like ECHO may obscure meaningful internal heterogeneity rather than improve interpretability. These results highlight the need for alternative frameworks that account for regional, socioeconomic, and environmental complexity beyond binary or ordinal codes.

This study has several limitations. Most notably, in our bootstrap analysis, we matched ECHO participants to county‐level PTB percentages from aligned time periods (2017–2019 or 2020–2022), ensuring temporal comparability. However, the HRSA estimates represent cumulative percentages over each 3‐year window and may smooth over important within‐period fluctuations in PTB risk due to the COVID‐19 pandemic. Studies report early declines in PTB rates in 2020 [41, 42] and subsequent increases linked to maternal infection through 2023 [43], which may not be fully captured in the aggregated estimates. As such, while our matching is time‐aligned, the source data may obscure short‐term variation in PTB risk.

Additionally, county‐level birth data in the HRSA file were obtained through a data use agreement that suppresses values in counties with few births. Because such counties are often rural, the empirical Bayesian smoothing applied to derive PTB percentages may disproportionately affect rural estimates [23, 44]. As a result, comparisons between ECHO and national PTB percentages may understate true variation in rural areas.

Next, differences in the measurement of race and ethnicity between the ECHO Cohort and the US Census affect our sociodemographic comparisons. ECHO survey instruments vary by cohort and may not align with the standardized race/ethnicity categories used in the ACS. Moreover, measurement error in ACS race/ethnicity estimates may be cause for concern, including a reported 411% increase in the “two or more races” category between 2019 and 2021 [45]. Such discrepancies likely affect the comparability of race/ethnicity distributions and may introduce bias into post‐stratification weights that rely on those benchmarks.

Finally, due to limitations in identifying exact home locations of participants from one cohort site in the West, we were unable to directly match county‐level PTB proportions to those participants. We instead matched state‐level PTB percentages for 2017–2019 and 2020–2022 using the unweighted mean restricted to the eight counties where those participants were recruited. These estimates include both rural and urban counties, potentially masking some rural–urban differences in PTB prevalence proportions.

## 5 Conclusion

This study underscores several structural limitations of rural–urban stratification in population health research. First, rural–urban classifications can combine substantively different places into a single category, blending variation in local social, economic, and environmental conditions under a shared geographic label. Second, binary classifications risk masking other critical drivers of health outcomes, such as economic disadvantage, physical barriers to healthcare access, and disproportionate environmental burdens. Third, we found that two predominantly rural cohort sites strongly influence PTB prevalence percentages, highlighting how cohort site composition can distort patterns of health outcomes across the broader cohort study. Finally, the relatively small size of the rural population in the study sample introduced asymmetry in precision and power, limiting the ability to generalize findings or detect structural effects. Together, these findings suggest that rural–urban stratification should be used with caution. We recommend that future research prioritize context‐aware stratification strategies and incorporate structural indicators to more accurately assess disparities and population‐level generalizability.

The key strength of this study is that it evaluated routine stratification by rural–urban codes to critically examine how classification schemes shape observed patterns in PTB. By demonstrating that the direction and magnitude of rural–urban differences vary by scheme, we challenge the assumption that rural–urban categories are stable or analytically neutral. The use of post‐stratification weights to align the ECHO Cohort with national sociodemographics foregrounds generalizability as a core analytic goal, while the leave‐one‐out sensitivity analysis underscores how rural–urban comparisons can be skewed by cohort composition.

Here, we highlight how the context of individual cohort sites can shape, and in some instances distort, the inferences drawn from stratified analyses. Rather than advocating for a single rural–urban classification scheme, we argue that caution is warranted when using rural–urban codes as analytic strata without taking strides to account for other contextual factors that may influence birth outcomes.

## Conflicts of Interest

The authors declare no conflicts of interest.

## Funding

The content is solely the responsibility of the authors and does not necessarily represent the official views of the National Institutes of Health. Research reported in this publication was supported by the Environmental influences on Child Health Outcomes (ECHO) Program, Office of the Director, National Institutes of Health, under Award Numbers U2COD023375 (Coordinating Center), U24OD023382 (Data Analysis Center), U24OD023319 with co‐funding from the Office of Behavioral and Social Science Research (Measurement Core), U24OD035523 (Lab Core), ES0266542 (HHEAR), U24ES026539 (HHEAR Barbara O'Brien), U2CES026533 (HHEAR Lisa Peterson), U2CES026542 (HHEAR Patrick Parsons, Kannan Kurunthacalam), U2CES030859 (HHEAR Manish Arora), U2CES030857 (HHEAR Timothy R. Fennell, Susan J. Sumner, Xiuxia Du), U2CES026555 (HHEAR Susan L. Teitelbaum), U2CES026561 (HHEAR Robert O. Wright), U2CES030851 (HHEAR Heather M. Stapleton, P. Lee Ferguson), UG3/UH3OD023251 (Akram Alshawabkeh), UH3OD023320 and UG3OD035546 (Judy Aschner), UH3OD023332 (Clancy Blair, Leonardo Trasande), UG3/UH3OD023253 (Carlos Camargo), UG3/UH3OD023248 and UG3OD035526 (Dana Dabelea), UG3/UH3OD023313 (Daphne Koinis Mitchell), UH3OD023328 (Cristiane Duarte), UH3OD023318 (Anne Dunlop), UG3/UH3OD023279 (Amy Elliott), UG3/UH3OD023289 (Assiamira Ferrara), UG3/UH3OD023282 (James Gern), UH3OD023287 (Carrie Breton), UG3/UH3OD023365 (Irva Hertz‐Picciotto), UG3/UH3OD023244 (Alison Hipwell), UG3/UH3OD023275 (Margaret Karagas), UH3OD023271 and UG3OD035528 (Catherine Karr), UH3OD023347 (Barry Lester), UG3/UH3OD023389 (Leslie Leve), UG3/UH3OD023344 (Debra MacKenzie), UH3OD023268 (Scott Weiss), UG3/UH3OD023288 (Cynthia McEvoy), UG3/UH3OD023342 (Kristen Lyall), UG3/UH3OD023349 (Thomas O'Connor), UH3OD023286 and UG3OD035533 (Emily Oken), UG3/UH3OD023348 (Mike O'Shea), UG3/UH3OD023285 (Jean Kerver), UG3/UH3OD023290 (Julie Herbstman), UG3/UH3OD023272 (Susan Schantz), UG3/UH3OD023249 (Joseph Stanford), UG3/UH3OD023305 (Leonardo Trasande), UG3/UH3OD023337 (Rosalind Wright), UG3OD035508 (Sheela Sathyanarayana), UG3OD035509 (Anne Marie Singh), UG3OD035513 and UG3OD035532 (Annemarie Stroustrup), UG3OD035516 and UG3OD035517 (Tina Hartert), UG3OD035518 (Jennifer Straughen), UG3OD035519 (Qi Zhao), UG3OD035521 (Katherine Rivera‐Spoljaric), UG3OD035527 (Emily S Barrett), UG3OD035540 (Monique Marie Hedderson), UG3OD035543 (Kelly J Hunt), UG3OD035537 (Sunni L Mumford), UG3OD035529 (Hong‐Ngoc Nguyen), UG3OD035542 (Hudson Santos), UG3OD035550 (Rebecca Schmidt), UG3OD035536 (Jonathan Slaughter), UG3OD035544 (Kristina Whitworth); NIMH R21MH140292 (Nissa Towe‐Goodman).

**Role of Funder**: The sponsor, NIH, participated in the overall design and implementation of the ECHO Program, which was funded as a cooperative agreement between NIH and grant awardees. The sponsor approved the Steering Committee‐developed ECHO protocol and its amendments including COVID‐19 measures. The sponsor had no access to the central database, which was housed at the ECHO Data Analysis Center. Data management and site monitoring were performed by the ECHO Data Analysis Center and Coordinating Center. All analyses for scientific publication were performed by the study statistician, independently of the sponsor. The lead author wrote all drafts of the manuscript and made revisions based on co‐authors and the ECHO Publication Committee (a subcommittee of the ECHO Operations Committee) feedback without input from the sponsor. The study sponsor did not review or approve the manuscript for submission to the journal.

## Supporting information




**Figure S1**. Flowchart showing inclusion and exclusion criteria. Abbreviations: ECHO, Environmental influences on Child Health Outcomes.
**Figure S2**. Average difference in 2017–2022 county‐level PTB rates by rural–urban classification (ECHO minus HRSA). Abbreviations: ECHO, Environmental influences on Child Health Outcomes; FPL, federal poverty level; HRSA, Health Resources and Services Administration; NCHS‐URC, National Center for Health Statistics Urban–Rural Codes; RUCC, Rural–Urban Continuum Codes; UIC, Urban Influence Codes. Notes: Stratified by 130% federal poverty level. Results from 10,000 bootstrap samples with replacement, post‐stratified using sample weights for race/ethnicity and educational attainment.
**Figure S3**. Average difference in 2017–2022 county‐level PTB rates by urban–rural classification stratified by census region (ECHO minus HRSA). Abbreviations: ECHO, Environmental influences on Child Health Outcomes; HRSA, Health Resources and Services Administration; NCHS‐URC, National Center for Health Statistics Urban–Rural Codes; RUCC, Rural–Urban Continuum Codes; UIC, Urban Influence Codes. Note: Results from 10,000 bootstrap samples with replacement, post‐stratified using sample weights for race/ethnicity and educational attainment.
**Table S1**. Proportion of eligible participants from ECHO Cohort (*n* = 65,794) with high‐quality geocodes stratified by rural and urban using 5 classification schemes.
**Table S2**. Effect estimates from sensitivity analysis sampling ECHO observations by pregnancy compared to sampling by child using post‐stratified sampling weights.

## Data Availability

Select de‐identified data from the ECHO Program are available through NICHD's Data and Specimen Hub (DASH). Information on study data not available on DASH, such as some Indigenous datasets, can be found on the ECHO study DASH webpage.
