# Supplementary material for: Challenges Associated With Rural‐Urban Stratification for Generalizing Birth Outcomes: Insights From the ECHO Cohort
Source: J Rural Health. 2026 May 12;42:e70163. doi: 10.1111/jrh.70163 (PMC13162180; doi:10.1111/jrh.70163)
Supplement: Supplementary file 1 — Figure S1. Flowchart showing inclusion and exclusion criteria. Abbreviations: ECHO, Environmental influences on Child Health Outcomes. Figure S2. Average difference in 2017–2022 county‐level PTB rates by rural–urban classification (ECHO minus HRSA). Abbreviations: ECHO, Environmental influences on Child Health Outcomes; FPL, federal poverty level; HRSA, Health Resources and Services Administration; NCHS‐URC, National Center for Health Statistics Urban–Rural Codes; RUCC, Rural–Urban Continuum Codes; UIC, Urban Influence Codes. Notes: Stratified by 130% federal poverty level. Results from 10,000 bootstrap samples with replacement, post‐stratified using sample weights for race/ethnicity and educational attainment. Figure S3. Average difference in 2017–2022 county‐level PTB rates by urban–rural classification stratified by census region (ECHO minus HRSA). Abbreviations: ECHO, Environmental influences on Child Health Outcomes; HRSA, Health Resources and Services Administration; NCHS‐URC, National Center for Health Statistics Urban–Rural Codes; RUCC, Rural–Urban Continuum Codes; UIC, Urban Influence Codes. Note: Results from 10,000 bootstrap samples with replacement, post‐stratified using sample weights for race/ethnicity and educational attainment. Table S1. Proportion of eligible participants from ECHO Cohort (n = 65,794) with high‐quality geocodes stratified by rural and urban using 5 classification schemes. Table S2. Effect estimates from sensitivity analysis sampling ECHO observations by pregnancy compared to sampling by child using post‐stratified sampling weights. [file JRH-42-0-s001.docx]

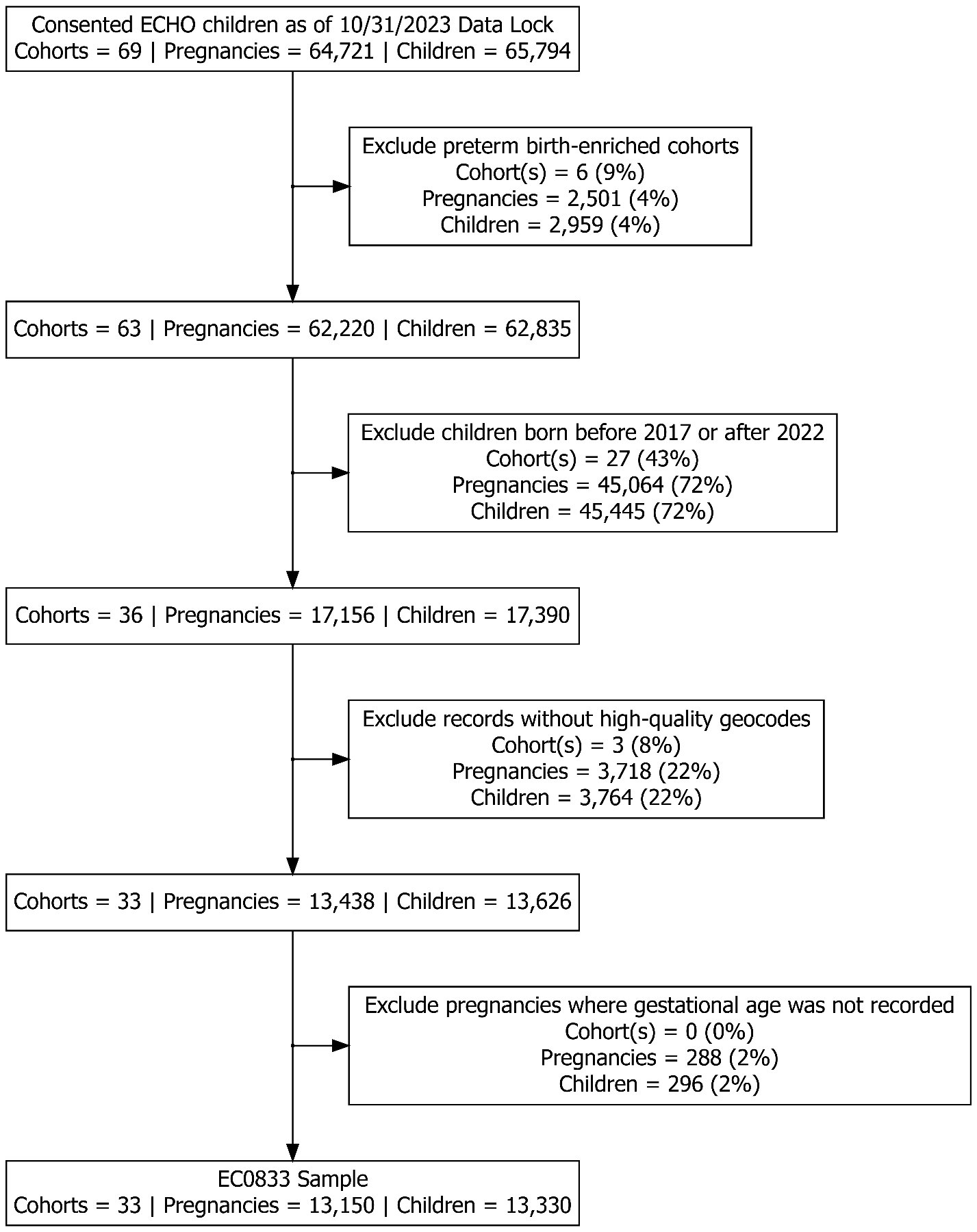


Supplemental Figure S1. Flowchart showing inclusion and exclusion criteria.

Abbreviations: ECHO, Environmental influences on Child Health Outcomes.

| 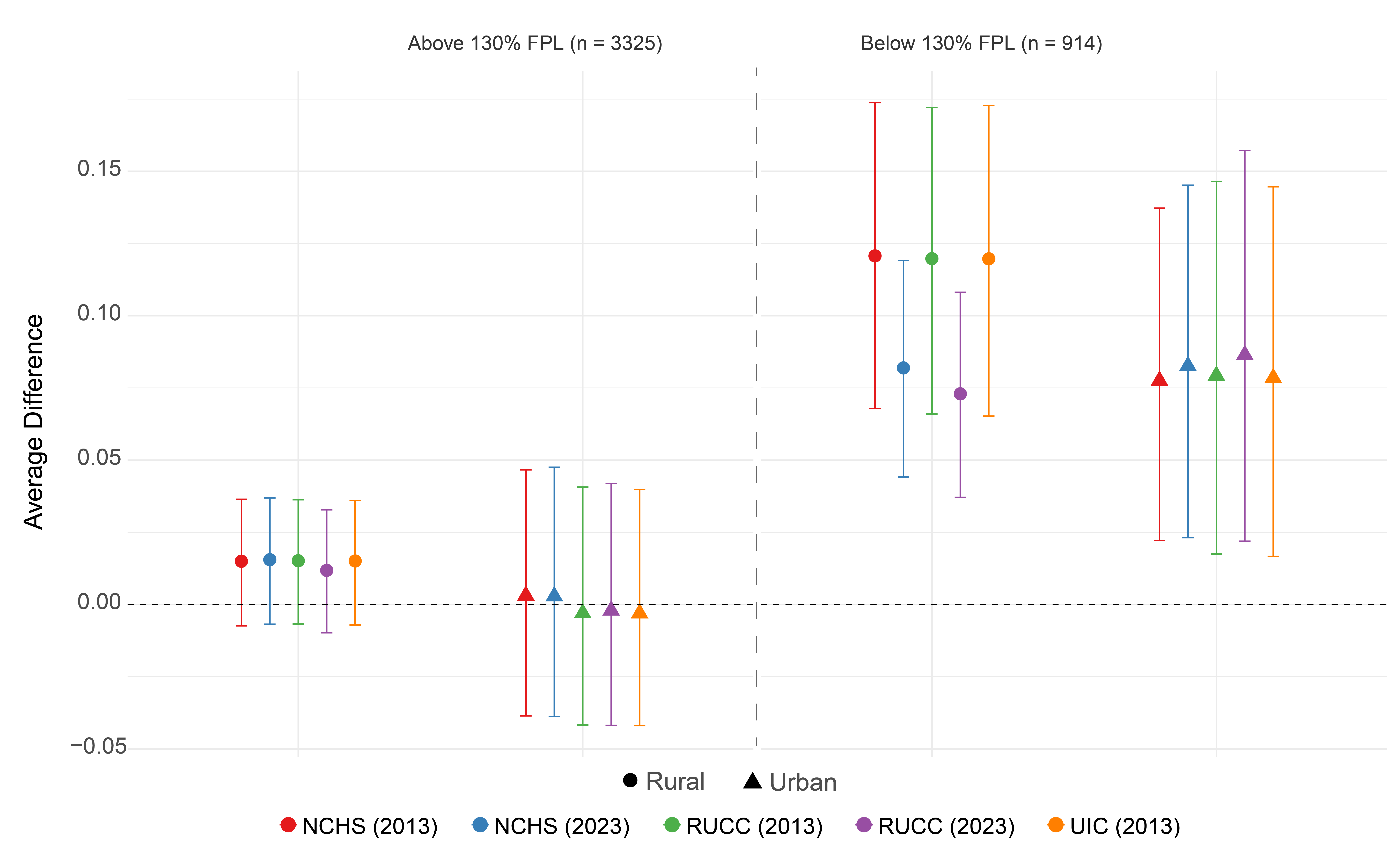 |
| --- |
| Supplemental Figure S2. Average difference in 2017–2022 county-level PTB rates by rural-urban classification (ECHO minus HRSA).  Abbreviations: ECHO, Environmental influences on Child Health Outcomes; FPL, federal poverty level; HRSA, Health Resources and Services Administration; NCHS-URC, National Center for Health Statistics Urban-Rural Codes; RUCC, Rural-Urban Continuum Codes; UIC, Urban Influence Codes.  Notes: Stratified by 130% federal poverty level. Results from 10,000 bootstrap samples with replacement, post-stratified using sample weights for race/ethnicity and educational attainment. |

| 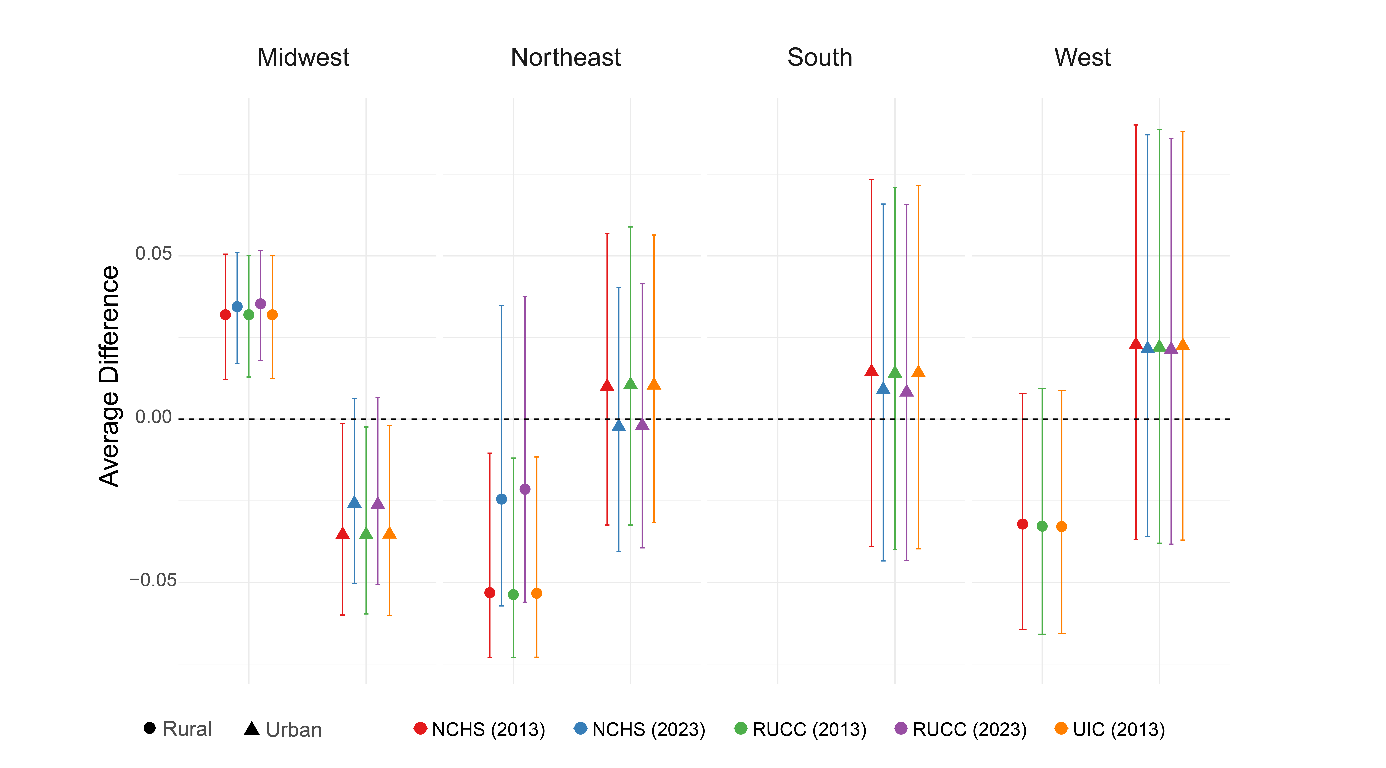 |
| --- |
| Supplemental Figure S3. Average difference in 2017–2022 county-level PTB rates by urban-rural classification stratified by census region (ECHO minus HRSA).  Abbreviations: ECHO, Environmental influences on Child Health Outcomes; HRSA, Health Resources and Services Administration; NCHS-URC, National Center for Health Statistics Urban-Rural Codes; RUCC, Rural-Urban Continuum Codes; UIC, Urban Influence Codes.  Note: Results from 10,000 bootstrap samples with replacement, post-stratified using sample weights for race/ethnicity and educational attainment. |

| Table S1. Proportion of eligible participants from ECHO Cohort (n = 65,794) with high-quality geocodes stratified by rural and urban using 5 classification schemes. | | | | | |
| --- | --- | --- | --- | --- | --- |
|  | RUCC (2013) | RUCC (2023) | UIC (2013) | NCHS (2013) | NCHS (2023) |
| Urban | 0.959 | 0.96 | 0.959 | 0.969 | 0.969 |
| Rural | 0.974 | 0.97 | 0.974 | 0.975 | 0.973 |

| Table S2. Effect estimates from sensitivity analysis sampling ECHO observations by pregnancy compared to sampling by child using post-stratified sampling weights. | | | | | | | |
| --- | --- | --- | --- | --- | --- | --- | --- |
|  |  | *Main model (child sampling)* | | | *Sensitivity analysis (pregnancy sampling)* | | |
| **RU Code** | **R/U** | **Mean** | **Min** | **Max** | **Mean** | **Min** | **Max** |
| RUCC 2013 | Urban | -0.0130 | -0.0270 | 0.0007 | -0.0181 | -0.0324 | -0.0041 |
|  | Rural | -0.0135 | -0.0250 | -0.0022 | -0.0170 | -0.0285 | -0.0054 |
| RUCC 2023 | Urban | -0.0133 | -0.0275 | 0.0005 | -0.0188 | -0.0330 | -0.0050 |
|  | Rural | -0.0110 | -0.0225 | 0.0005 | -0.0134 | -0.0249 | -0.0019 |
| UIC 2013 | Urban | -0.0130 | -0.0268 | 0.0007 | -0.0181 | -0.0321 | -0.0041 |
|  | Rural | -0.0136 | -0.0251 | -0.0023 | -0.0170 | -0.0286 | -0.0056 |
| NCHS 2013 | Urban | -0.0152 | -0.0291 | -0.0016 | -0.0208 | -0.0353 | -0.0065 |
|  | Rural | -0.0142 | -0.0262 | -0.0026 | -0.0177 | -0.0294 | -0.0058 |
| NCHS 2023 | Urban | -0.0151 | -0.0291 | -0.0015 | -0.0209 | -0.0352 | -0.0067 |
|  | Rural | -0.0146 | -0.0264 | -0.0029 | -0.0171 | -0.0291 | -0.0049 |
